# The association of sun sensitivity, sun protective behaviors and depression in both genders: a study based on the U.S. population

**DOI:** 10.3389/fpubh.2025.1505941

**Published:** 2025-04-02

**Authors:** Jipeng Zhang, Hongfei Mo, Xinruncheng Zhong, Rui Feng

**Affiliations:** ^1^School of Physical Education (Main Campus), Zhengzhou University, Zhengzhou, China; ^2^School of Public Health, Fudan University, Shanghai, China; ^3^Shiquan County Hospital, Ankang, China

**Keywords:** sun sensitivity, sun protective behaviors, depression, gender differences, NHANES

## Abstract

**Objective:**

The purpose of this study was to analyze the association between sun sensitivity, common sun protective behaviors (stay in shade, wear long sleeves, use sunscreen) and depression, respectively, in both genders, after adjusted for each other as confounders.

**Materials and methods:**

Data from the National Health and Nutrition Examination Survey 2017–2018 cycle were aggregated. Sun sensitivity and sun protective behaviors were assessed through the dermatology questionnaire. Depression was assessed through the 9-item Patient Health Questionnaire, with a score > 4 as the cutoff point. Gender specific logistic regressions were carried out to analyze the association between sun sensitivity, sun protective behaviors and depression.

**Results:**

A sample of 2,605 participants (mean age 39.99 ± 11.57 years) was analyzed, including 1,227 (47.1%) males and 1,378 (52.9%) females. No association between sun sensitivity and depression was observed. In the sample, stay in shade (OR = 1.27, 95% CI: 1.03–1.57) was positively associated with depression, use sunscreen (OR = 0.69, 95% CI: 0.53–0.90) was negatively associated with depression. Gender specific regressions showed no associations between sun protective behaviors and depression in males; both wear long sleeves (OR = 0.65, 95% CI: 0.42–0.99) and use sunscreen (OR = 0.71, 95% CI: 0.52–0.97) were negatively associated with depression in females.

**Conclusion:**

No association between sun sensitivity and depression. Stay in shade was positively associated with depression, while use sunscreen was negatively associated. Gender differences were observed: no association between sun protective behaviors and depression in males; wear long sleeves and use sunscreen may be negatively associated with depression in females.

## 1 Background

Depression is one of the most prevalent mental disorders worldwide. It affects millions of people, leading to severe economic and social consequences, and may increase the risk of other diseases and even death. The World Health Organization (WHO) reported that approximately 322 million people suffer from depression, making it one of the leading causes of disability worldwide (1). The burden of depression extends beyond an individual’s personal suffering, impacting families, communities, and society at large, making it a severe public health burden.

Sun exposure has been suggested as a protective factor against depression, and both vitamin D and endorphins may play a role in this association. Sun exposure stimulates the skin’s production of vitamin D, which plays a crucial role in brain function and mood regulation. It has been shown that vitamin D deficiency is associated with an increased risk of depression across all age range (2). In addition, exposure to sunlight stimulates the release of a neurotransmitter known as endorphin, which serves to ameliorate emotional states and alleviate pain (3).

While sun exposure is beneficial, there are individuals who are unable to safely expose themselves to the sun due to skin sensitivities or conditions such as sun sensitivity. These individuals may need to take extra precautions, such as wearing protective clothing or using sunscreen. Additionally, there is still a significant portion of the population who hold the belief that sun exposure is harmful (4) and engage in sun avoiding behaviors to minimize their time in the sun. Therefore, we speculate there is a possibility that avoiding sun exposure may affect vitamin D synthesis and the release of certain mood-positive neurotransmitters, which could potentially impact mental health, and trigger depression.

There is another of fact that depression exhibits differences between male and female genders. Studies have shown that women generally experience higher rates of depression compared to men, possibly attributed to biological, hormonal, and psychosocial factors (5, 6). Regarding sun protection behaviors, research suggests that women tend to exhibit higher levels of sun protection compared to men. Studies have found that women are more likely to use sunscreen, seek shade, and wear protective clothing when exposed to the sun (7, 8). We suggest that gender differences in depression are likely to be associated with different sun protection behaviors in the two genders.

Based on the above views, we suggest that sun sensitivity and sun protective behaviors are likely to be associated with depression, and that there may be gender differences in this association. However, studies exploring the association between sun sensitivity or sun protective behaviors and depression risk are limited, and further research is needed. Therefore, we designed this study to analyze the association between sun sensitivity, common sun protective behaviors (stay in shade, wear long sleeves, use sunscreen) and depression, respectively, in both genders, after adjusted for each other as confounders.

## 2 Materials and methods

### 2.1 Study sample

**Figure 1:**
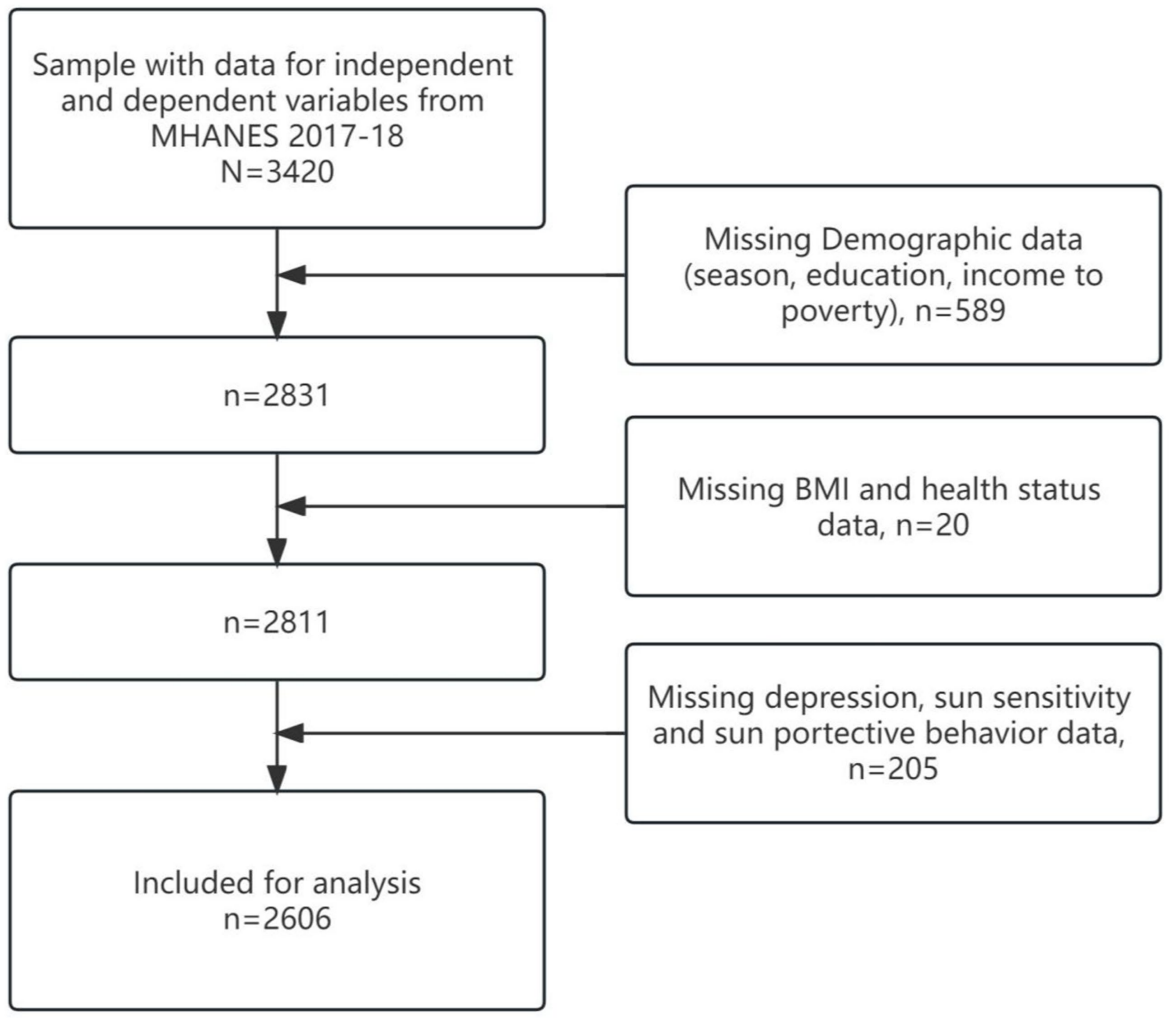
Flow chart of subject selection.

The sample for this study was obtained from the National Health and Nutrition Examination Survey (NHANES) 2017–18 cycle. The NHANES is a population based cross-sectional survey designed to collect information on the health and nutrition of the U.S. household population. The survey is conducted on a two-year cycle and consists of both household interviews and health assessments. NHANES protocols and secondary analyses of the data are approved by the National Center for Health Statistics Research (NCHS) Ethics Review Board, and all adult participants provide written notification of consent. NHANES uses a stratified multi-stage sampling design to obtain a representative sample of U.S. residents. The sampling plan consisted of four stages: selection of primary sampling units, counties or neighboring group counties; selection of units within counties; selection of dwelling units and selection of sample persons within dwelling units. Sampling and exclusions for the present study are presented in the flow chart below (see Figure 1).

### 2.2 Sun sensitivity and sun protective behaviors assessment

Sun sensitivity and sun protective behaviors were the independent variables in this study. They were assessed by the Dermatology Questionnaire (DEQ), which provides personal interview data on sun exposure and sun protective behavior. The DEQ were eligible for participants aged 20–59 years, and was asked, in the home, by trained interviewers using the Computer-Assisted Personal Interview (CAPI) system.

Sun sensitivity was assessed using one question “If after several months of not being in the sun, you then went out in the sun without sunscreen or protective clothing for a half hour, which one of these would happen to your skin?” (response options: get a severe sunburn with blisters/a severe sunburn for a few days with peeling/mildly burned with some tanning/turning darker without a sunburn/nothing would happen in half an hour). Those who responded “get a severe sunburn with blisters” and “a severe sunburn for a few days with peeling” were classed as “sun sensitive.” The rest were classed as “not sensitive.”

Sun protective behaviors were categorized into “Stay in shade,” “Wear long sleeves,” and “Use sunscreen.” They were assessed using question “When you go outside on a very sunny day, for more than 1 h, how often do you stay in the shade?,” “Wear a long sleeved shirt? Would you say…,” “Use sunscreen? Would you say…,” respectively (response options: always/most of the time/sometimes/rarely/never). Those who responded “always” and “most of the time” were classed as “yes (have that kind of sun protective behavior),” while the rest were classed as “no.”

### 2.3 Depression assessment

Depression was the dependent variable in the analysis of this study. It was assessed by the Patient Health Questionnaire (PHQ-9), a 9-item depression screening instrument used to assess the frequency with which participants experienced depressive symptoms in the past 2 weeks. The items in the questionnaire were asked at the Mobile Examination Center (MEC) by trained interviewers using a CAPI system. For each item, points ranging from 0 to 3 represent the response categories of “not at all,” “a few days,” “more than half the days,” and “almost every day,” respectively. Those with complete responses to the symptom questions could calculate a total score ranging from 0 to 27, and scores >4 were considered to have depression (9). In this study, participants with PHQ-9 scores of 0–4 were categorized as the “non-depression group” and scores of 5–27 were categorized as the “depression group.”

### 2.4 Covariates

Covariates included age, gender, race, season of exam, military service, education, marital status, household members, income to poverty ratio (PIR), body mass index (BMI), and current health status. Among them, age was not categorized. Gender (male/female), race (Mexican American/other Hispanic/non-Hispanic White/non-Hispanic Black/non-Hispanic Asian/other race), season of exam (November–April/May–October), military service (yes/no), education (less than 9th grade/9–11th grade/high school/some college/college or above), marital status (married/widowed/divorced/separated/never married/living with partner), household members (1/2/3/4/5/6/7 or above) and current health status (excellent/very good/good/fair/poor) were categorized using the original NHANES categorization (10). PIR was categorized into impoverished (<1.3) and moderate income (≥1.3) (11). BMI was defined as weight in kg/(height in meters)^2^ and categorized into standard categories: underweight (≤18.9 kg/m^2^), normal weight (19.0–24.9 kg/m^2^), overweight (25.0–29.9 kg/m^2^), and obese (≥30.0 kg/m^2^) (12).

### 2.5 Statistical analyses

First, we used Microsoft Excel 2010 to organize the raw data: we merged the data tables for independent variables, dependent variables, and covariates, and we excluded missing values.

Second, in order to compare the differences between the “depression group” and the “non-depression group,” we conducted analyses of variance of covariates and independent variables using Statistical Product and Service Solutions (SPSS) 28.0: we used *t*-test or *z*-test for normally distributed continuous variables, rank-sum test for skewed continuous variables, and chi-square test for categorical variables.

Third, to analyze the association between sun sensitivity, sun protective behavior and depression, we conducted logistic regression analyses using SPSS 28.0. We also progressively adjusted for confounders through the regression models. In analyzing the association between sun sensitivity and depression, we developed a 2-step regression model: Model I: Original model, no adjustment for any confounding variables; Model II: Adjusted for the independent variable in the last model plus gender, race, education, marital status, household members, income status, BMI category, current health status. In analyzing the association between sun protective behaviors and depression, we added 2 models to the previous 2-step model: Model III: Adjusted for the independent variable in the last model plus sunlight sensitivity; Model IV: Adjusted for the independent variable in the last model plus the other two sun protective behaviors.

Finally, we divided males and females into two subgroups, and analyzed the association between sun sensitivity, sun protective behaviors and depression, respectively. The regression models were used as described in the previous paragraph, except that gender was not adjusted in the second model. To ensure the accuracy and reliability of the results and control for errors arising from multiple comparisons, the Bonferroni correction method was employed. The original significance level was set at *α* = 0.05. Since a total of 4 × 2 = 8 comparisons were made, the adjusted significance level was calculated as *α*’ = 0.05/8 = 0.00625. In the gender subgroup analyses, the statistical *p*-values obtained were compared with the adjusted significance level α’. A variable was considered statistically significantly associated with the dependent variable in the corresponding subgroup if its *p*-value was less than α’.

## 3 Results

### 3.1 Demographic characteristics

The present study included a total of 2,606 participants from the NHANES 2017–18 cycles who have completed data on depression, sun sensitivity, sun protective behaviors, gender, and other covariates. Sample’s mean age was 39.99 ± 11.57 years at the time of examination, with 1,227 (47.1%) males and 1,378 (52.9%) females. There were 680 (26.10%) individuals in the depression group and 1925 (73.90%) in the non-depression group. Statistically significant differences were observed in all covariates except for season of exam and military service, and in all independent variables (all *p* < 0.05) (see Table 1).

**Table 1:** Sample demographic characteristics, by depression.

Characteristics (*n*%)	Total *N* = 2,605	Depression group 680 (26.10)	Non-depression group 1925 (73.90)	Statistic	*P*
Weighted Number (n)	*N* = 134,727,421	*n* = 33,527,331	*n* = 101,200,089		
Age (year, Mean ± SD)	39.99 ± 11.57	40.59 ± 11.77	39.78 ± 11.49	*t* = −1.565	0.118
Gender				*χ*^2^ = 15.669	<0.001
Male	1,227 (47.1)	276 (40.59)	951 (49.40)		
Female	1,378 (52.9)	404 (59.41)	974 (50.60)		
Race				*χ*^2^ = 53.942	<0.001
Mexican American	377 (14.47)	97 (14.26)	280 (14.55)		
Other Hispanic	214 (8.21)	56 (8.24)	158 (8.21)		
Non-Hispanic White	869 (33.36)	262 (38.53)	607 (31.53)		
Non-Hispanic Black	581 (22.3)	149 (21.91)	432 (22.44)		
Non-Hispanic Asian	399 (15.32)	53 (7.79)	346 (17.97)		
Other Race	165 (6.33)	63 (9.26)	102 (5.30)		
Season of Exam				*χ*^2^ = 0.217	0.642
November–April	1,288 (49.44)	331 (48.68)	957 (49.71)		
May–October	1,317 (50.56)	349 (51.32)	968 (50.29)		
Military Service				*χ*^2^ = 0.597	0.440
Yes	142 (5.45)	41 (6.03)	101 (5.25)		
No	2,463 (94.55)	639 (93.97)	1824 (94.75)		
Education				*χ*^2^ = 51.525	<0.001
Less than 9th grade	131 (5.03)	44 (6.47)	87 (4.52)		
9-11th grade	266 (10.21)	90 (13.24)	176 (9.14)		
High school	613 (23.53)	168 (24.71)	445 (23.12)		
Some college	917 (35.2)	268 (39.41)	649 (33.71)		
College or above	678 (26.03)	110 (16.18)	568 (29.51)		
Marital statues				*χ*^2^ = 68.128	<0.001
Married	1,238 (47.52)	244 (35.88)	994 (51.64)		
Widowed	40 (1.54)	21 (3.09)	19 (0.99)		
Divorced	239 (9.17)	90 (13.24)	149 (7.74)		
Separated	94 (3.61)	35 (5.15)	59 (3.06)		
Never married	652 (25.03)	190 (27.94)	462 (24.00)		
Living with partner	342 (13.13)	100 (14.71)	242 (12.57)		
Household members				*χ*^2^ = 31.825	<0.001
1	219 (8.41)	82 (12.06)	137 (7.12)		
2	611 (23.45)	188 (27.65)	423 (21.97)		
3	576 (22.11)	142 (20.88)	434 (22.55)		
4	519 (19.92)	116 (17.06)	403 (20.94)		
5	367 (14.09)	77 (11.32)	290 (15.06)		
6	182 (6.99)	42 (6.18)	140 (7.27)		
7 or above	131 (5.03)	33 (4.85)	98 (5.09)		
IPR (mean)	2.16 (1.15–4.16)	1.52 (0.82–3.04)	2.47 (1.29–4.62)	*Z* = −10.186	<0.001
Income status				*χ*^2^ = 78.020	<0.001
Impoverished	1826 (70.1)	386 (56.76)	1,440 (74.81)		
Moderate income	779 (29.9)	294 (43.24)	485 (25.19)		
BMI (kg/m^2^, Mean ± SD)	30.06 ± 7.89	31.24 ± 8.44	29.64 ± 7.64	*t* = −4.346	<0.001
BMI category				*χ*^2^ = 28.118	<0.001
Underweight	72 (2.76)	23 (3.38)	49 (2.55)		
Normal weight	653 (25.07)	139 (20.44)	514 (26.70)		
Overweight	749 (28.75)	168 (24.71)	581 (30.18)		
Obese	1,131 (43.42)	350 (51.47)	781 (40.57)		
Current Health Status				*χ*^2^ = 278.450	<0.001
Excellent	268 (10.29)	38 (5.59)	230 (11.95)		
Very good	642 (24.64)	89 (13.09)	553 (28.73)		
Good	1,098 (42.15)	252 (37.06)	846 (43.95)		
Fair	530 (20.35)	252 (37.06)	278 (14.44)		
Poor	67 (2.57)	49 (7.21)	18 (0.94)		
Sunlight sensitivity				*χ*^2^ = 6.970	0.008
Sun sensitive	2,323 (89.17)	588 (86.47)	1735 (90.13)		
Not sensitive	282 (10.83)	92 (13.53)	190 (9.87)		
Stay in shade				*χ*^2^ = 9.180	0.002
Yes	1,691 (64.91)	409 (60.15)	1,282 (66.60)		
No	914 (35.09)	271 (39.85)	643 (33.40)		
Wear long sleeves				*χ*^2^ = 4.232	0.040
Yes	2,237 (85.87)	600 (88.24)	1,637 (85.04)		
No	368 (14.13)	80 (11.76)	288 (14.96)		
Use sunscreen				*χ*^2^ = 12.030	<0.001
Yes	2046 (78.54)	566 (83.24)	1,480 (76.88)		
No	559 (21.46)	114 (16.76)	445 (23.12)		

### 3.2 Logistic regression analyses

In the sample, after adjusted for confounding variables, the association between sun sensitivity and depression was not statistically significant (*p* = 0.678). Of the 3 common sun-protective behaviors, stay in shade showed a positive association with depression, OR (95% CI): 1.27 (1.03–1.57), *p* = 0.024; Use sunscreen showed a negative association with depression, OR (95% CI): 0.69 (0.53–0.90), *p* = 0.006; Wear long sleeves was not statistically significant (*p* = 0.155) (see Table 2).

**Table 2:** Association of sun sensitivity, sun protective behaviors and depression, respectively.

Predictors/Models	*β*	S.E	Z	*P*	OR (95% CI)
Sun sensitivity					
Model I	0.36	0.14	2.63	0.009	1.43 (1.10–1.86)
Model II	0.07	0.16	0.42	0.678	1.07 (0.78–1.46)
Stay in shade					
Model I	0.28	0.09	3.03	0.002	1.32 (1.10–1.58)
Model II	0.19	0.11	1.81	0.070	1.21 (0.98–1.49)
Model III	0.19	0.11	1.78	0.075	1.21 (0.98–1.48)
Model IV	0.24	0.11	2.26	**0.024**	**1.27 (1.03–1.57)**
Wear long sleeves					
Model I	−0.28	0.14	−2.05	0.040	0.76 (0.58–0.99)
Model II	−0.21	0.15	−1.38	0.166	0.81 (0.60–1.09)
Model III	−0.22	0.15	−1.40	0.161	0.81 (0.60–1.09)
Model IV	−0.22	0.16	−1.42	0.155	0.80 (0.59–1.09)
Use sunscreen					
Model I	−0.40	0.12	−3.45	<0.001	0.67 (0.53–0.84)
Model II	−0.34	0.13	−2.54	0.011	0.71 (0.55–0.92)
Model III	−0.35	0.13	−2.61	0.009	0.70 (0.54–0.92)
Model IV	−0.37	0.14	−2.73	**0.006**	**0.69 (0.53–0.90)**

OR, Odd Ratio. Model I: Original model, no adjustment for any confounding variables. Model II: Adjusted for the independent variable in the last model plus gender, race, education, marital status, household members, income status, BMI category, current health status. Model III: Adjusted for the independent variable in the last model plus sunlight sensitivity. Model IV: Adjusted for the independent variable in the last model plus the other two sun protective behaviors. Same as below. Boldface indicates that the results are still statistically significant after adjusting for all covariates, i.e., *p* < 0.05.

In males, after adjusted for confounding variables, the association of all predictors with depression were not statistically significant (*p* > 0.00625) (see Table 3).

**Table 3:** Association of sun sensitivity, sun protective behaviors and depression, in males.

Predictors/Models	*β*	S.E	*Z*	*P*	OR (95% CI)
Sun sensitivity					
Model I	0.55	0.23	2.42	0.016	1.73 (1.11–2.70)
Model V	0.43	0.26	1.66	0.096	1.54 (0.93–2.57)
Stay in shade					
Model I	0.34	0.15	2.23	0.026	1.40 (1.04–1.89)
Model V	0.29	0.17	1.66	0.096	1.33 (0.95–1.86)
Model III	0.26	0.17	1.53	0.125	1.30 (0.93–1.82)
Model IV	0.29	0.17	1.68	0.093	1.34 (0.95–1.88)
Wear long sleeves					
Model I	−0.21	0.21	−0.99	0.320	0.81 (0.54–1.22)
Model V	0.02	0.23	0.10	0.917	1.02 (0.65–1.61)
Model III	0.00	0.23	0.00	1.000	1.00 (0.64–1.57)
Model IV	0.01	0.23	0.02	0.981	1.01 (0.64–1.59)
Use sunscreen					
Model I	−0.50	0.25	−2.03	0.042	0.60 (0.37–0.98)
Model V	−0.32	0.27	−1.18	0.237	0.72 (0.42–1.24)
Model III	−0.39	0.28	−1.40	0.161	0.68 (0.39–1.17)
Model IV	−0.44	0.28	−1.55	0.122	0.65 (0.37–1.12)

Model V: Adjusted for the independent variable in the last model plus race, education, marital status, household members, poverty state, BMI category, health status. Same as below.

In females, after adjusted for confounding variables, the association between sun sensitivity and three sun protection behaviors, as well as depression, was not statistically significant (*p* > 0.00625). However, Of the 3 common sun-protective behaviors, wear long sleeves showed a negative association with depression, OR (95% CI): 0.65 (0.42–0.99), *p* = 0.043; use sunscreen showed a negative association with depression, OR (95% CI): 0.71 (0.52–0.97), *p* = 0.032. The OR value within the 95% confidence interval did not include 1, suggesting that there might be an association between women’s use of long-sleeved clothes and sun protection sleeves for sun protection and depression. However, due to the strictness of the multiple testing corrections, the *p*-value was not significant (see Table 4).

**Table 4:** Association of sun sensitivity, sun protective behaviors and depression, in females.

Predictors/Models	*β*	S.E	*Z*	*P*	OR (95% CI)
Sun sensitivity					
Model I	0.19	0.17	1.11	0.269	1.21 (0.86–1.68)
Model V	−0.17	0.20	−0.84	0.404	0.84 (0.56–1.26)
Stay in shade					
Model I	0.14	0.12	1.16	0.245	1.15 (0.91–1.45)
Model V	0.13	0.14	0.95	0.342	1.14 (0.87–1.49)
Model VI	0.14	0.14	1.04	0.299	1.15 (0.88–1.51)
Model VII	0.23	0.14	1.61	0.107	1.25 (0.95–1.65)
Wear long sleeves					
Model I	−0.34	0.18	−1.90	0.057	0.71 (0.50–1.01)
Model V	−0.43	0.21	−2.00	0.046	0.65 (0.43–0.99)
Model VI	−0.42	0.21	−1.98	0.048	0.66 (0.43–0.99)
Model VII	−0.44	0.22	−2.02	**0.043**	**0.65 (0.42–0.99)**
Use sunscreen					
Model I	−0.54	0.14	−3.99	<0.001	0.58 (0.44–0.76)
Model V	−0.34	0.16	−2.20	0.028	0.71 (0.52–0.96)
Model VI	−0.33	0.16	−2.12	0.034	0.72 (0.53–0.97)
Model VII	−0.34	0.16	−2.14	**0.032**	**0.71 (0.52–0.97)**

Boldface indicates that the results are still statistically significant after adjusting for all covariates, i.e., *p* < 0.05.

## 4 Discussion

The results of the present study suggest that there are no statistically significant association between sun sensitivity and depression. There is an association between sun protective behaviors and depression, with gender-specific variations observed in this relationship. We discuss these findings below.

### 4.1 No association between sun sensitivity and depression

The results showed no association between sun sensitivity and depression, either in the sample or in both genders. Among relevant studies, we did not find any reports of sun sensitivity (photosensitivity, ultraviolet sensitivity) and depression associations. One possibility is that sun sensitivity is weakly associated with depression and that our small sample size was not sufficient to analyze this association. Another possibility is that sun sensitivity and depression are two factors that exist independently of each other, there is no direct correlation between the two factors. In fact, sun sensitivity was more important acting as a covariate in this study: there may be a higher correlation or causality between sun sensitivity and sun protective behaviors, and adjusting for sun sensitivity is necessary when analyzing the association between sun protective behaviors and depression.

### 4.2 Sun protective behavior differences in depression

The results showed that stay in shade was a potential risk factor for depression, while use sunscreen was a potential protective factor. There are very limited explanations directly related to this result, which we consider to be a new finding. We suggest that stay in shade actually reduces sun exposure and limits the opportunity to obtain the potential benefits of sunlight. Several studies have demonstrated an association between sun exposure and improved mood and mental well-being (13, 14). Linos et al. reported that sun protection behaviors among Americans may elevate the risk of vitamin D deficiency (15). Sun exposure stimulates the production of vitamin D, which is associated with decreased risk of depression (16). By avoiding sun exposure and hiding in the shade, individuals may miss out on the positive effects of sunlight, potentially increasing the risk of depression. Furthermore, vitamin D deficiency may exert negative impacts on mental health, including anxiety, behavioral issues, and psychological stress (17, 18), which may also increase the likelihood of depression. Conversely, applying sunscreen protects the skin from harmful ultraviolet radiation while still enjoying the benefits of sunshine. Using sunscreen essentially reflects a optimistic attitude toward life, and optimism have been shown to be associated with a reduced risk of depression (19, 20). Sunscreen use also reflects the user’s endorsement of sun exposure, which makes it likely that they will actively seek out the sun and thus receive more benefits than others. In addition, sun exposure after sunscreen application not only provides emotional benefits, but also prevents skin damage, which has been shown to be associated with negative mental health outcomes (21).

### 4.3 Gender differences in the association

The results identified a gender difference in the association between sun protective behaviors and depression: there is no association in males, while there is an association in females. Evidence supporting our result are also very limited. We speculate that sun protection behaviors may indirectly impact female hormone secretion, with fluctuations in these hormones potentially influencing women’s emotional states. Conversely, the male endocrine system may exhibit less sensitivity to sun protection behaviors. Males might also display reduced sensitivity to vitamin D deficiency due to variations in physiological structure, dietary habits, or lifestyles, coupled with the chemical similarity between vitamin D and sex hormones (22). Consequently, no significant correlation is observed between sun protection behaviors and depression in males.

Women tend to be more concerned about their appearance and skin condition, with good skin potentially enhancing their self-confidence and well-being, thereby reducing the risk of depression (23). Men may have lower concerns about skin condition, rendering the correlation between sun protection behaviors and self-esteem or mental health less evident. Sunlight promotes the secretion of neurotransmitters like serotonin and dopamine (24). By engaging in appropriate sun protection (e.g., wearing long sleeves and using sunscreen) while enjoying sunlight, women can protect their skin and potentially maintain a good emotional state.

Due to sociocultural influences, women generally exhibit greater concern for skin health and appearance (25). This sociocultural pressure may impact women’s mental health, and appropriate sun protection behaviors may be perceived as a coping mechanism. Conversely, men are typically not affected by such sociocultural pressures. Additionally, women may be more inclined to maintain mental health through social activities. Proper sun protection enables them to maintain skin health during outdoor activities, thereby facilitating more active social engagement and enhancing mental well-being.

### 4.4 Limitations

This study has several limitations. (1) The data used were surveyed in 2017–2018, which is rather old and does not reflect the current situation. (2) The adjustment of confounding variables can be improved, such as adjusting more factors of diseases related to depression. (3) Vitamin D, a key factor in the current research hypothesis, was not presented in the study. The relationship between sun protection behaviors, vitamin D, and depression, as well as whether vitamin D serves as a mediator in the association between sun protection behaviors and depression, or whether it should be considered as a covariate, remains to be investigated. (4) This cross-sectional study unveils an association between sun protection behaviors and depression, yet it does not establish causality.

## 5 Conclusion

There was no significant association between sun sensitivity and depression. Among sun protective behaviors, stay in shade was positively associated with depression, while use sunscreen was negatively associated. There are gender differences in the association between sun protective behaviors and depression: no association in males; wear long sleeves and use sunscreen may be negatively associated with depression in females.

## Data Availability

Publicly available datasets were analyzed in this study. This data can be found: NHANES Questionnaires, Datasets, and Related Documentation (cdc.gov).
